# Successful pregnancy following gastric sleeve surgery for long-duration primary infertility: A case report

**DOI:** 10.1016/j.ijscr.2023.108100

**Published:** 2023-04-07

**Authors:** Amal Yaseen Zaman

**Affiliations:** Obstetrics and Gynecology, Taibah University, College of Medicine, Department of Obstetrics and Gynecology, Al Madinah Munawara, Saudi Arabia

**Keywords:** Sleeve surgery, Infertility, Pregnancy, Obesity, Case report

## Abstract

**Introduction and importance:**

Morbid obesity negatively affects patients' life quality in several ways. One of the major obesity-related problems is getting pregnant, even when using assisted reproductive technology. Obesity contributes to anovulation and menstrual irregularities, with a lower conception rate, a lower response to fertility treatment, poor implantation, low-quality oocytes, and miscarriages. Managing morbid obesity and then assessing the pregnancy outcome is crucial.

**Case presentation:**

We reported a case of a 42-year-old woman who had primary infertility for 26 years, polycystic ovary syndrome (PCOS), and a body mass index (BMI) of 51. She was able to get pregnant after having bariatric sleeve surgery, which brought her BMI down to 27. She had a successful pregnancy and live birth by Intrauterine insemination (IUI) from the first trial.

**Clinical discussion:**

Patients with morbid obesity (BMI 35) and obesity-related health problems have often turned to bariatric surgery as their first line of treatment. Extremely overweight females with PCOS and infertility may benefit more from bariatric surgery.

**Conclusion:**

Extremely overweight females with PCOS and infertility may benefit more from bariatric surgery, such as laparoscopic sleeve gastrectomy, than rather than only a healthier lifestyle change. Larger studies that assess the effect of bariatric surgeries in highly morbid females with PCOS are needed.

## Highlights

• Morbid obesity negatively affects patients' life quality in several ways.
• Polycystic ovary syndrome is associated with several negative impacts on pregnancy.
• Obesity promotes PCOS and impacts treatment efficacy, including infertility.
• Extremely overweight females with PCOS and infertility may benefit more from bariatric surgery.

## 1 Introduction

Worldwide, the rate of obesity is highly rising. The World Health Organization (WHO) reports that 39 % of the global population has a BMI of 25 or higher, and 13 % has a BMI of 30 or higher [1]. Numerous comorbidities, such as elevated blood pressure, arthritis, cancer, and type 2 diabetes, have long been associated with obesity [1], [2], [3], [4], [5]. Overweight or obese pregnant women have higher chances of developing gestational diabetes, preeclampsia [6], [7], [8] spontaneous miscarriage [9], large for gestational-age children, and fetal abnormalities [10]. Epigenetic modifications also increase the risk of hypertension, diabetes, and cardiovascular diseases in the descendants of obese mothers [11]. Additionally, polycystic ovary syndrome (PCOS) is associated with several negative impacts on pregnancy such as the increased risk of premature births. Androgen excess is one of many potential etiologic factors in preterm delivery through cervix remodeling [12]. An increase in inflammatory mediators has been also linked to a hyperandrogenic state, which may play a role in cervix shortening or preterm birth [13], [14].

Between three and 10 % of women of childbearing age have PCOS, more than any other endocrine disorder [1], [15]. Ovarian health and fertility are affected by several factors, but obesity is a major one. Obesity promotes PCOS and substantially affects the efficacy of treating all components of the condition, including infertility. The chance of miscarriage, congenital abnormalities, and other pregnancy-related issues is increased, and it affects the likelihood of conceiving and responding to fertility treatment [2]. Most people who struggle with obesity have a predisposition due to genetics, a poor diet, and a lack of physical activity, which in turn exacerbates metabolic disturbances.

Moreover, one reason why overweight women produce low gonadotropins is that their bodies convert more androgens to estrogens in the periphery. Insulin resistance and hyperinsulinemia, both are prevalent in obese women, causing hyperandrogenism [16]. When leptin levels rise, the levels of sex hormone-binding globulin, growth hormone, and insulin-like growth factor-binding proteins all fall. Consequently, the neuro-regulation of the HPG axis (hypothalamic, pituitary, and gonadal) is disrupted. These alterations may shed light on why ovulatory function and, by extension, fertility, are declining. Therefore, several factors may explain the link between obesity and infertility as higher insulin resistance and leptin levels, as well as hyperandrogenemia. Similarly, anovulation, alterations in adipokine levels and the HPG axis, and steroidogenesis all have an impact on the reproductive system in obese women [17].

Bariatric surgery (BS) is sometimes suggested if all other attempts to reduce weight have failed. It has positive effects on heart function [18], and fertility [19]. It is one of the most effective methods for losing weight and reducing the risk of many comorbidities [20], [21]. International recommendations according to American College of Obstetricians and Gynecologists (ACOG) guidelines suggested that BS should be considered if a patient's BMI is over 40 kg/m^2^ or if the BMI is between 35 kg/m^2^ and 40 kg/m^2^ but with significant comorbidities [22].

This case report has been reported in line with the SCARE Criteria [23]. In our study, we present a case of a 42-year-old woman who, despite having PCOS and a BMI of 51, finally became pregnant after 26 years of trying following a sleeve surgery.

## 2 Presentation of case

A 42-year-old Saudi woman with an initial BMI of 51 kg/m^2^ and significant impairment of quality of life was considered for bariatric surgery (sleeve gastric surgery) due to the failure of intensive lifestyle modification to maintain weight loss.

Her medical history contained hypothyroidism, which was controlled by thyroxin 100 mic for 20 years. She also has had type two diabetes mellitus for 20 years, which was managed with Glucophage 750 mg twice daily but was not well controlled. Furthermore, she has had bronchial asthma since childhood and she is using Ventolin and steroid inhalers. Regarding her fertility, she had many cycles of ovulation induction by clomiphene citrate and injectable gonadotropins with failed pregnancy even with reaching a good size of the follicle of 16 mm or more. The patient suffered from long-duration infertility for about 26 years.

The past gynecological history includes irregular menstruation and prolonged periods (8–10 days). The patient underwent successful sleeve gastric surgery on July 13th, 2018. After the sleeve surgery, the patient's condition improved. BMI reduced to 27 kg/m^2^. Also, the menstrual cycle became regular with nearly four days of bleeding every 28–30 days of average amount. Her blood glucose is controlled using Glucophage same dosage form and her hemoglobin A1c was 5.2 and no more attacks of asthma since surgery. The thyroid function also improved with a TSH level of 2.4 and normal levels of free T3 and T4. Her husband is a 45-year-old non-smoker, medically and surgically free.

Hysterosalpingography (HSG) study was done and showed a normal uterine cavity and patent tubes with free spillage before the intrauterine insemination (IUI) procedure. On day two of the cycle March 8th, 2020, using transvaginal ultrasound, the patient has an endometrial thickness of 4 mm, and both adnexa were free of cysts. Then, stimulation was started by Menopure 75 IU for 11 days and she was followed up by serial transvaginal ultrasound until she had a follicle of 18 mm in size and endometrial thickness of 11 mm then, IUI was done on March 22nd, 2020.

Table 1 shows the semen analysis after washing. After insemination, luteal phase support started by using cyclogest 400 mg twice daily, Duphaston 10 mg twice daily, and aspirin 80 mg. The patient continued her regular medications which were thyroxin 100 mic and Glucophage 750 mg twice daily.

**Table 1:** Semen analysis.

		Normal
● Volume (ml)	0.8 ml	1.5–5 ml
● Count (10^6^/ml)	5 × 10^6^/ml	>15 × 10^6^/ml
● Motility (%)	50 %	≥40 %
● Normal morphology (%)	30 %	≥4 %

After 14 days, the pregnancy test was positive with a β-HCG level of 233. At the 7th week of gestation, the transabdominal ultrasound revealed a single viable intrauterine fetus.

**Fig. 1:**
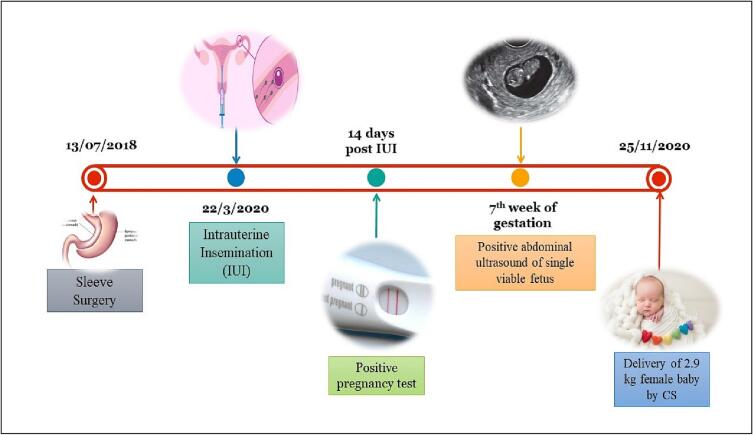
Stepwise representation of the case.

The patient did very well during her pregnancy without any complications, but her thyroxin level is increased to 200 mics for two days and 100 mics for 5 days per week. The patient had an elective cesarean section on November 25th, 2020, and had a female baby weighing 2.9 kg. The patient's journey from sleeve surgery to delivery is summarized in Fig. 1.

## 3 Discussion

The British Fertility Society recommends a BMI of <30 for pregnancy. For some people, a weight loss of just 5–10 % of their body weight is all it takes to improve their fertility and metabolic health [6]. Over the last decade, minimally invasive bariatric surgery has become increasingly popular. The worldwide obesity crisis undoubtedly plays a role in the care of obese women with PCOS. Although the exact mechanism by which bariatric surgery improves PCOS is uncertain, a meta-analysis of the effect of bariatric surgery on PCOS by Skubleny et al. revealed a 40 % improvement in monthly irregularities and a 30 % reduction in hirsutism [7], also Bhandari et al. proved that the decrease in hirsutism in six months of follow-up was significant, as 74.6 % of the population had a complete resolution of hirsutism at the end of follow-up [24].

It is recommended for a patient with a BMI of 35 kg/m^2^ to have bariatric surgery, even if they have a severe metabolic disorder [11]. Patients with a lower BMI (<50 kg/m^2^) had a higher percent expected weight loss (EWL) at three months, whereas super-obese patients (BMI > 50 kg/m^2^) had a higher percent EWL after six months [25]. The studied woman underwent sleeve gastrectomy rather than another procedure since it was indicated given her BMI of 51 and a diabetic condition. At the time of starting IUI, the patient had a BMI of 27.

In addition to the well-established health benefits and quality-of-life enhancements associated with bariatric surgery, individuals often report dramatic improvements in their sexual performance. There are currently suggestions to wait up to 18 months after surgery before trying to conceive. There is, however, insufficient data to conclude that pregnancy in the first postoperative year is hazardous. However, obesity may not be the primary cause of hyperandrogenemia and anovulation, as some obese women are fertile despite their weight. Hyperinsulinemia and insulin resistance, together with hyperandrogenemia and alterations in steroidogenesis, are the root causes of obesity. Numerous effects of insulin on steroidogenesis have been observed [26]. Hyperandrogenemia is a hallmark of PCOS, and infertility was formerly thought to be the major recognized complication of PCOS. Moreover, losing weight can help PCOS patients lower their androgen levels and enhance their insulin sensitivity [27], [28]. After many years of infertility due to obesity and PCOS, the patient after surgery lost huge weight, got pregnant, and her condition was stable during the pregnancy and delivery without any complications affecting the woman or her female baby.

In summary, while the benefits of significant weight loss for fertility and PCOS are well-known, bariatric surgery may offer a viable option for highly morbid females struggling with infertility and PCOS symptoms. Further research is needed to fully assess the effects of bariatric surgery on fertility and PCOS, and larger studies that assess the effects of bariatric surgeries in highly morbid females with PCOS are needed to produce more valid results.

## 4 Conclusions

Patients with morbid obesity (BMI 35) and obesity-related health problems have often turned to bariatric surgery as their first line of treatment. Extremely overweight females with PCOS and infertility may benefit more from bariatric surgery, such as laparoscopic sleeve gastrectomy, than rather than only a healthier lifestyle change. Larger studies that assess the effect of bariatric surgeries in highly morbid females with PCOS are needed to produce more valid results.

## Ethical approval

Ethical approval obtained.

## Funding

None.

## Author contribution

This is a single author work.

## Guarantor

Dr. Amal Yaseen Zaman.

## Consent

Informed consent was obtained from the patient for presentation of the details of this case.

## Declaration of competing interest

There are no conflicts of interest to report.
